# Effect of sputum quality and role of Xpert^®^ MTB/ RIF assay for detection of smear-negative pulmonary tuberculosis in same-day diagnosis strategy in Addis Ababa, Ethiopia

**DOI:** 10.4102/ajlm.v11i1.1671

**Published:** 2022-08-31

**Authors:** Waganeh Sinshaw, Abebaw Kebede, Adane Bitew, Mengistu Tadesse, Zemedu Mehamed, Ayinalem Alemu, Bazezew Yenew, Misikir Amare, Biniyam Dagne, Getu Diriba, Ephrem Tesfaye, Dinka F. Gamtesa, Yeshiwork Abebaw, Helina Molallign Mollalign, Getachew Seid, Muluwork Getahun

**Affiliations:** 1 Tuberculosis Research Unit/National Tuberculosis Reference Laboratory, Ethiopian Public Health Institute, Addis Ababa, Ethiopia; 2 Department of Microbiology, Immunology and Parasitology, School of Medicine, Addis Ababa University, Addis Ababa, Ethiopia; 3 Department of Medical Laboratory Science, College of Health Sciences, Addis Ababa University, Addis Ababa, Ethiopia; 4 Department of Microbial, Cellular, and Molecular Biology, College of Natural and Computational Sciences, Addis Ababa University, Addis Ababa, Ethiopia

**Keywords:** smear-negative pulmonary tuberculosis, Xpert® MTB/RIF test, sputum quality, tuberculosis culture, diagnostic performance

## Abstract

**Background:**

There is limited information on the performance of the Xpert^®^ MTB/RIF test for diagnosis of smear-negative pulmonary tuberculosis (SNPT) and rifampicin resistance (RR) in the same-day diagnosis approach. The effects of sputum quality and other factors affecting the Xpert performance are also under-investigated.

**Objective:**

This study aimed to determine the performance of the Xpert^®^ MTB/RIF test for detection of SNPT and RR in the same-day diagnosis strategy and the effect of sputum quality and other factors on its performance.

**Methods:**

A cross-sectional study was conducted from August 2017 to January 2018 across 16 health facilities in Addis Ababa, Ethiopia. Two spot sputum samples were collected from 418 presumptive SNPT patients, tested with Xpert® MTB/RIF, then compared to tuberculosis culture. Additionally, culture isolates were tested for RR by BACTEC MGIT™ 960 drug susceptibility testing (DST) and MTBDRplus version 2.

**Results:**

The Xpert^®^ MTB/RIF test detected 24 (5.7%) SNPT cases, with a sensitivity of 92.3% (75.9% – 97.9%) and specificity of 99.2% (97.8% – 99.7%) compared with tuberculosis culture. Xpert^®^ MTB/RIF also detected three (11.58%) RR strains with 100.0% concordance with BACTEC MGIT™ 960 DST and MTBDRplus results. Three blood-stained SNPT samples were positive by Xpert (30.0%), which was 6.9 times higher compared to salivary sputum (odds ratio: 6.9, 95% confidence interval: 1.36–34.96, *p* = 0.020).

**Conclusion:**

The performance of the Xpert^®^ MTB/RIF to detect SNPT and RR in same-day diagnosis is high. However, SNPT positivity varies among sputum qualities, and good sample collection is necessary for better test performance.

## Introduction

Smear-negative pulmonary tuberculosis (SNPT) occurs when a presumptive pulmonary tuberculosis (PTB) patient tests negative by acid-fast bacilli microscopy but tests positive by more accurate diagnostic techniques.^1^ It is one of the most problematic issues in tuberculosis diagnosis.^2^ Patients with SNPT contributed 17.3% – 41.0% of community tuberculosis transmission in Vancouver, Canada, from January 1995 to March 1999^3^ and 13.0% in the Netherlands from 1996 to 2004.^4^ Also, 55.4% of Belarus tuberculosis cases were smear-negative but culture-positive in 2012.^5^ In Ethiopia, bacteriologically confirmed SNPT prevalence was reported as high as 23.9% in 2007.^6^

Although SNPT is assumed to be less contagious and have lower mortality compared to smear-positive tuberculosis, 50.0% – 71.0% of SNPT patients develop active tuberculosis disease.^3,4^ These SNPT patients also harbour a high proportion of drug-resistant tuberculosis strains. The prevalence of smear-negative multidrug-resistant (MDR) tuberculosis among presumptive PTB patients enrolled with other patients was 47.0% in Belarus in 2012^5^ and 11.5% in Ethiopia August 2017 to January 2018.^7^ Mortality due to SNPT is also substantial, especially among the immunocompromised. In Mozambique, the mortality of HIV co-infected patients reached 55.8%.^8^ Thus, early detection with sensitive diagnostic tools that simultaneously detect drug-resistant tuberculosis is critical.

However, diagnosis of SNPT and smear-negative drug-resistant tuberculosis is challenging in low- and middle-income countries (LMICs) like Ethiopia,^9^ mainly due to a lack of sensitive diagnostic tools. In most LMICs, smear microscopy, which has low sensitivity, is still the first line of diagnosis. The Xpert^®^ MTB/RIF test is a cartridge-based, fully automated DNA testing platform that, in less than 2 h, simultaneously detects tuberculosis and mutations conferring rifampicin resistance (RR).^10^ The technology detects RR using five probes targeting mutations in the rpoB region of the *Mycobacterium tuberculosis* genome^11,12^: rpoB mutations are responsible for RR in over 99.5% of RR strains.^10^

The Xpert^®^ MTB/RIF test currently resolves the challenges of initial smear microscopy in tuberculosis case detection, which improves the clinical management of tuberculosis cases.^13,14^ In addition, the Xpert^®^ MTB/RIF is advantageous over smear microscopy and *M. tuberculosis* culture by its higher sensitivity, simultaneous detection of RR, shorter turnaround time (TAT) (2 h), and a minimal safety requirement.^15^ By comparison, culture-based RR confirmation takes more than two weeks to get results.^16^

Nevertheless, various factors impact the Xpert^®^ MTB/RIF’s performance, such as the type of specimen,^17^ sputum quality,^18,19,20^ sample collection and diagnosis strategy,^21,22,23^ and clinical characteristics of patients.^17,24,25^ Since 2012, more than 314 GeneXpert^®^ instruments have been installed in Ethiopia. Also, the diagnostic strategy changed from spot-morning-spot, which required three samples collected over two days, to same-day diagnosis (spot-spot diagnosis), which requires two samples collected on the same day.^26^

However, information is lacking on the performance of the Xpert^®^ MTB/RIF test to diagnose SNPT, smear-negative RR tuberculosis, the effects of sputum quality and other factors in the same-day tuberculosis diagnosis approach in Addis Ababa, Ethiopia. Therefore, this study aimed to determine the performance of the Xpert^®^ MTB/RIF test to diagnose SNPT and RR against conventional tuberculosis culture and drug susceptibility testing (DST) in the spot-spot diagnostic strategy. It also intended to determine the SNPT positivity in sputum samples with varying quality and the effect of sputum quality and other factors on Xpert^®^ MTB/RIF test performance.

## Methods

### Ethical considerations

The study was reviewed and approved by the Institutional Review Board of the Ethiopian Public Health Institute (ref. no. EPHI/613/535) and the Departmental Research and Ethics Review Committee of the Department of Medical Laboratory Science, Addis Ababa University (ref. no. MLS/364/17). Written informed consent from the participants and assent from the guardians of participants less than 18 years of age were obtained. Permission was requested with a legal letter and received from participanting health facilities. Unique patient identifiers were used instead of patient names to conceal patient identity. The laboratory testing results and other patient information were locked. Laboratory results were reported to the clinicians who ordered the test for further patient management.

### Study setting and design

This cross-sectional study was conducted from August 2017 to January 2018 in Addis Ababa, Ethiopia. Addis Ababa is the federal capital of Ethiopia and has a population of more than three million.^27^ The study was conducted in 16 systematically selected governmental and private directly observed treatment short-course tuberculosis sites. These sites participate in blind rechecking, and most participate in the on-site supervision programme by the Addis Ababa City Administration health research and laboratory service.

### Data collection

Socio-demographic information, clinical presentations, comorbidities, and other factors were collected using a structured questionnaire by trained clinicians through interviews. The questionnaire was piloted following the on-site training of the data collectors.

### Patient enrollment

A total of 418 presumptive SNPT patients who were negative for two consecutive spot sputum smears were enrolled. Adults and children with presumptive PTB who visited the sites during the study period were eligible. However, those taking anti-tuberculosis drugs for more than one week or unable to submit two spot sputum samples were excluded.

Trained laboratorians gave patients detailed instructions on sputum collection. Each presumptive SNPT patient collected two sputum spot ≥ 3 mL samples into sterile 50 mL falcon tubes. Sputum samples were transported with a triple packaging system to the National Tuberculosis Reference Laboratory (NTRL) of the Ethiopian Public Health Institute (EPHI) for laboratory investigation. Sputum samples were stored at 2 °C – 8 °C until transported by triple packaging system for safety, with a thermometer inside for temperature monitoring. The transportation from study sites to NTRL takes less than one hour since it is within the city. Samples were submitted to NTRL within a maximum of two days. Macroscopic sputum quality was evaluated and categorised as blood-stained, purulent, mucoid, and salivary based on the Global Laboratory Initiative (GLI) guidance.^28^

### Laboratory investigations

The two consecutive spot sputum samples collected from each patient were pooled into one and homogenised. Afterwards, each sputum pool was split into two; one for Xpert^®^ MTB/RIF test (Cepheid, Sunnyvale, California, United States) and the other for culture testing on BACTEC™ MGIT™ 960 System (Becton-Dickinson and Company, Sparks, Maryland, United States) and in laboratory-made Lowenstein Jensen (LJ). Drug susceptibility testing (phenotypic and genotypic) was performed in the NTRL of EPHI, as explained in Sinshaw et al., 2019.^7^

### Quality assurance and quality control

The sterility and performance of LJ media-manufactured in-house were verified before use for testing using controls by randomly selecting some prepared LJ medium, putting it in the LJ incubator and monitoring for any contaminant growth. If no growth was observed within 56 days, it was considered as sterile or safe for use. Likewise, a new lot or batch of BACTEC™ MGIT™ 960 media was also verified. Each of the culture and identification procedures was performed in a certified Class II biosafety cabine. Preventive maintenance of the equipment, temperature monitoring, and instrument operation checks were performed. In each batch of sample cultured, reagent sterility and process contamination check control were incorporated based on the NTRL standard procedures. A proficiency testing scheme continuously monitored all study test methods. Also, the NTRL is International Organization for Standardization 15189 accredited by the Ethiopian National Accreditation Office for Xpert^®^ MTB/RIF.

### Data entry and analysis

Double data entry was perfomed on EpiData statistical software version 3.0 (EpiData Association, Odense, Denmark). The clean data were transferred to and analysed using IBM Statistical Package for Social Sciences software version 20.0 (Chicago, Illinois, United States). Data entry and cleaning were performed by the NTRL data manager. The characteristics of the study participants were analysed using descriptive statistics. The sensitivity and specificity of the Xpert^®^ MTB/RIF test were calculated at 95% confidence intervals (CI). Pearson chi-square, Fisher’s exact test, or binary logistic regression was used to determine the associations between dependent and independent variables; variables with a *p* ≤ 0.2 were selected for multivariable analysis. The strength of associations was measured by odds ratios, and a *p* < 0.05 was taken as statistically significant.

## Results

### Socio-demographic and clinical characteristics of study participants

Most of the participants (231; 55.3%) were female. The average participant age was 36 years (standard deviation [s.d.] ± 18). Most of the participants (257, 61.8%) had completed some schooling (Grades 1–12), while 112 (26.9%) were uneducated, and more than half of the participants (218; 52.3%) were married. Cough was the leading symptom (413; 98.8%), followed by fever (255; 61.2%). Of the 225 participants interviewed or laboratory tested, 57 (25.3%) were positive for HIV (Table 1 and Table 2).

**TABLE 1:** Socio-demographic characteristics of the participants and their association with SNPT detection by Xpert MTB/RIF test in Addis Ababa, Ethiopia, from August 2017 to January 2018.

Variables	Positive		Negative		*p*	Adjusted odds ratio		*p*
	*n*	%	*n*	%		Odds ratio	95% confidence interval	
**Sex**								
Male	19	10.20	168	89.80	0.006	3.850	1.290–11.44	0.015
Female	8	3.50	223	96.50		Ref	-	-
Total	27	6.50	391	93.50		-	-	-
**Age group**								
< 14	0	0.00	20	100. 00	0.225	-	-	-
15–24	7	7.40	87	92.60		-	-	-
25–34	12	9.60	113	90.40		-	-	-
35–44	5	8.20	56	91.80		-	-	-
45–54	1	1.90	52	98.10		-	-	-
> 54	2	3.10	63	96.90		-	-	-
Total	27	6.50	391	93.50		-	-	-
**Education**								
No formal education	7	6.20	105	93.80	0.050	Ref	-	-
Grade 1–7	4	3.40	112	96.60		0.006	0.000–0.19	0.003
Grade 8–12	10	7.10	131	92.90		0.011	0.000–0.30	0.008
> Grade 12	5	10.60	42	89.40		0.013	0.000–0.40	0.012
Total‡	26	6.25	390	93.75		-		-
**Marital status** †								
Single	13	8.20	146	91.80	0.530	-	-	-
Married	12	5.50	206	94.50		-	-	-
Divorced/widowed/separated	2	5.00	38	95.00		-	-	-
Total‡	27	6.50	390	93.50		-	-	-
**Work†**								
Farmer	1	3.20	30	96.80	0.006	Ref	-	-
Daily laborer	6	7.90	70	92.10		3.310	0.355–30.96	0.293
Housewife	1	1.10	86	98.90		0.610	0.026–14.42	0.760
Driver	0	0.00	10	100.00		-	-	-
Teacher	0	0.00	7	100.00		-	-	-
Student	0	0.00	46	100.00		-	-	-
Merchant	0	0.00	14	100.00		-	-	-
Health professional	1	33.30	2	66.70		38.300	1.077–1368	0.045
Government employee	2	5.70	33	94.30		1.551	0.100–23.40	0.750
Self-employed	7	11.70	53	88.30		5.250	0.563–48.90	0.150
Other	7	16.30	36	83.70		7.331	0.770–69.85	0.083
Total‡	25	6.10	387	93.90		-	-	-

† , One participant did not respond;

‡ , Six participants did not respond.

**TABLE 2:** Clinical manufestations and behavioural characteristics of the presumptive SNPT patients in Addis Ababa, Ethiopia, from August 2017 to January 2018.

Variables	Yes/no	Positive	%	Negative	%
**Clinical and behavioural characteristics**					
Cough	Yes	27	6.5	386	93.5
	No	0	0.0	5	100.0
Fever	Yes	20	7.8	235	92.2
	No	7	4.3	155	95.7
Chest pain	Yes	21	8.3	231	91.7
	No	6	3.6	160	96.4
Loss of appetite	Yes	22	9.1	221	90.9
	No	5	2.9	170	97.1
Weight loss	Yes	20	12.4	141	87.6
	No	7	2.7	249	97.3
Shortness of breath	Yes	14	2.7	120	89.6
	No	13	4.6	271	95.4
Joint pain	Yes	10	9.0	101	91.0
	No	17	5.5	290	94.5
Abdominal pain	Yes	2	4.0	48	96.0
	No	25	6.8	343	93.2
Swelling	Yes	1	5.6	17	94.4
	No	26	6.5	374	93.5
Night sweating	Yes	4	6.9	58	93.1
	No	23	6.1	355	93.9
Hemoptysis	Yes	4	13.8	25	86.2
	No	23	5.9	365	94.1
Shifting of trachea	Yes	0	0.0	5	100.0
	No	27	6.6	385	93.4
Abnormal breathing sound	Yes	6	6.2	90	93.8
	No	21	6.5	301	93.5
Dullness during percussion	Yes	4	11.8	30	88.2
	No	23	6.0	359	94.0
Tenderness	Yes	4	15.4	22	84.6
	No	23	5.9	367	94.1
HIV infection	Yes	3	5.3	54	94.7
	No	14	8.3	154	91.7
Migrant	Yes	3	27.3	8	72.7
	No	24	5.9	380	94.1
MDR TB contact	Yes	1	12.5	7	87.5
	No	26	6.4	381	93.6
Drug-susceptible TB contact	Yes	2	7.4	25	92.6
	No	25	6.4	363	93.6
Alcohol consumption	Yes, currently	9	13.2	59	86.8
	Yes, previously	2	3.6	53	96.4
	Not at all	16	5.4	279	94.6
Smokers	Yes, currently	2	15.4	11	84.6
	Yes, previously	2	15.4	11	84.6
	Not at all	23	6.0	361	94.0
Chewing chat	Yes, currently	3	12.0	22	88.0
	Yes, previously	5	15.6	27	84.4
	Not at all	19	5.3	342	94.7
**Treatment history†**					
New	-	24	6.5	345	93.5
Previously treated	-	3	6.1	46	93.9

MDR, multidrug-resistant.

† , Previously treated means patients who have taken anti-TB treatment in their life for 1 month or more.

### Sputum quality and performance characteristics of Xpert^®^ MTB/RIF test

Salivary sputum was the leading sample quality (147, 35.2%), while blood-stained sputum was the least (10; 2.4%) sputum quality submitted (Table 3). The majority of the sputum submitted (310; 74.3%) had 3 mL – 5 mL volume, while 16.8% had 6 mL – 7 mL and 8.9% had 8 mL – 9 mL volume.

**TABLE 3:** Positivity of Xpert MTB/RIF assay in different sputum sample qualities in Addis Ababa, Ethiopia, from August 2017 to January 2018.

Variables	Xpert MTB/RIF result				Chi-square (*p*)	Adjusted odds ratio	95% confidence interval	*p*
	Negative		Positive					
	*n*	%	*n*	%				
**Sputum quality**								
Purulent	108	92.3	9	7.7	0.013	1.39	0.500–3.88	0.520
Mucoid	137	95.8	6	4.2		0.71	0.232–15.00	0.540
Saliva	138	93.9	9	6.1		Reference	-	-
Blood-stained	7	70.0	3	30.0		6.90	1.360–34.96	0.020

**Total†**	**390**	**93.5**	**27**	**6.5**		**-**	**-**	**-**

† , Difference in expected total is because one sputum sample was not evaluated for macroscopic appearance.

The majority of the positive Xpert^®^ MTB/RIF tests, 14 (51.9%), were low and very low in bacillary load. There were 20 (4.8%) unsuccessful Xpert MTB/RIF results (sum of errors, invalids and no results). The most unsuccessful results were errors (15; 3.6%) (Table 4).

**TABLE 4:** Semi-quantitative bacilli DNA quantification and unsuccessful Xpert MTB/RIF test results in Addis Ababa, Ethiopia, from August 2017 to January 2018.

Variables	Frequency	%
**Bacilli DNA quantification by Xpert MTB/RIF test**		
High	5	18.52
Medium	8	29.63
Low	9	33.30
Very low	5	18.52
Total	27	100.00
**Unsuccessful Xpert MTB/RIF results**		
Error	15	3.60
Invalid	2	0.50
No result	3	0.70
Total	20	4.80

The error code 5007 was the most common (Table 5). Mucoid sputum accounted for seven (46.7%) errors, while purulent sputum accounted for five (33.3%). The other three (20.0%) errors were from salivary sputum.

**TABLE 5:** Xpert MTB/RIF test post-run analysis error results and possible causes in Addis Ababa, Ethiopia, from August 2017 to January 2018.

Error code	Error message (taken from cepheid user manual)	*n*	%	Origin of the error (taken from cepheid user manual)
5007	Probe check control failed and the test was stopped before amplification	12	2.9	Sputum viscosity or wrong sample volume, or cartridge reaction tube improperly filled, contains bubbles, or probe integrity issue detected
5017	Quality control 1 and quality control 2 probe check failed	1	0.24	Cartridge-related issue (based on cepheid information)
5011	Signal loss detected in the amplification curve for analyte (specimen processing control)	2	0.48	Loss of tube pressure because the cartridge tube is not airtight, or cartridge valve is not working correctly

*Source:* Adapted from World Health Organization. Xpert MTB/RIF implementation manual. Geneva: WHO, 2014; p. 1–35.

The Xpert^®^ MTB/RIF test detected three RR cases. Two of the RR cases were caused by probe E (codons 529–533) missing mutations and one by probe B (codons 511–518) missing mutation.

### Sputum *M. tuberculosis* positivity rate

*Mycobacterium tuberculosis* positivity rate was 6.1% for salivary sputum and 4.2% for mucoid sputum. However, the *M. tuberculosis* positivity rate was higher (30.0%) in blood-stained sputum. Blood-stained sputum was 6.9 times more *M. tuberculosis* positive than salivary sputum (95% CI: 1.36–34.96; odds ratio [OR]: 6.9; *p* = 0.02), while purulent sputum was the second most positive (7.7%) (Table 3).

### Detection of SNPT and RR by Xpert^®^ MTB/RIF test

**FIGURE 1:**
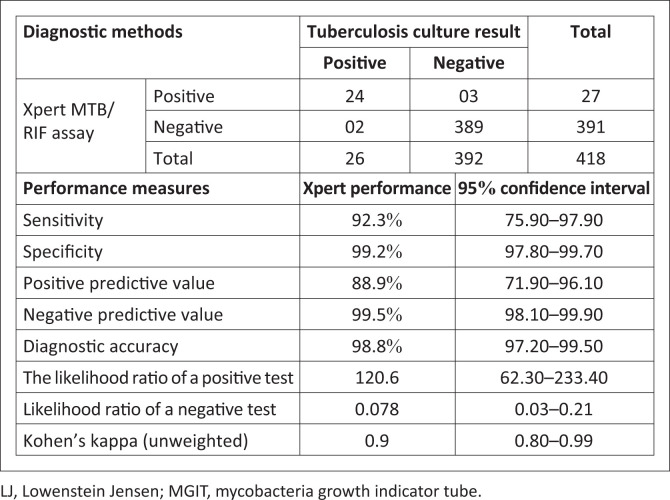
Xpert^®^ MTB/RIF assay performance reference to the MGIT and LJ *Mycobacterium tuberculosis* culture in Addis Ababa, Ethiopia, from August 2017 to January 2018.

Of the 418 smear-negative presumptive PTB patients enrolled in this study, 27 (6.5%; 95% CI: 4.10–8.81) were *M. tuberculosis* positive by Xpert^®^ MTB/RIF. On the other hand, 26 (6.4%; 95% CI: 4.00–8.74) were culture-positive. Twenty-four (5.7%; 95% CI: 3.51% – 7.97%) sputum samples were positive by both Xpert^®^ MTB/RIF and tuberculosis culture (LJ and MGIT) (Figure 1).

Five samples were discordantly positive by both methods; one sample was LJ culture-positive but MGIT culture-negative. On the other hand, four samples were MGIT culture-positive, two of which were LJ culture-negative, while the other two were LJ culture contaminated. Besides, of the two Xpert^®^ MTB/RIF negative culture-positive samples, one was MGIT negative and the other MGIT positive, but both were positive by LJ solid culture. Moreover, three sputum samples were Xpert^®^ MTB/RIF positive but culture-negative. Thus, the diagnostic sensitivity and specificity of the Xpert^®^ MTB/RIF test relative to tuberculosis culture was 92.3% (75.9% – 97.9%) and 99.2% (97.8% – 99.7%). The overall diagnostic accuracy was 98.8% (97.2% – 99.5%) (Figure 1).

Three (11.54%) RR SNPT cases were detected by the Xpert^®^ MTB/RIF test; two of the RR detected were from a new case and one from previously treated cases. These strains were later confirmed as MDR by genotype MTBDRplus version 2 and BACTEC™ MGIT™ 960 DST.

Most of the SNPT cases were detected from men (95% CI: 1.29–11.44; OR: 3.85; *p* = 0.015]) (Table 1). Patients with weight loss had 4.05 times more risk of being diagnosed with SNPT, in 95% CI (*p* = 0.007) compared to those without weight loss (Supplementary Table 1).

## Discussion

Smear microscopy is still a front-line tuberculosis diagnostic tool in developing countries like Ethiopia.^1,21,29^ However, because of its low sensitivity, smear microscopy misses many tuberculosis cases, resulting in delayed diagnosis and treatment initiation.^15,30,31^ Therefore, this study evaluated the effect of sputum quality and Xpert^®^ MTB/RIF performance for SNPT detection in same-day diagnosis.

Sputum quality has effects on the detection of PTB.^19,20,32,33^ However, many studies do not consider attributes of sputum quality in MTB testing by the Xpert^®^ MTB/RIF test.^34,35^ In the present study, the *M. tuberculosis* positivity by Xpert^®^ MTB/RIF significantly varied across sputum qualities. The majority of sputum, 147 (35.2%), submitted was salivary with a positivity rate of 6.1%. Although the blood-stained sample was the least submitted, the positivity was very high at 30.0%. In 95% CI, blood-stained samples showed 6.9 (*p* = 0.020) times more positive than salivary.

Many laboratories reject blood-stained sputum, assuming that it brings unreliable Xpert results due to polymerase chain reaction inhibition; however, this study revealed the highest SNPT positivity in blood-stained sputum. Contrary to the present study, a study showed that the Xpert result is only valid at less than 2.0% blood contamination of the sputa. If sputum contamination with blood is beyond 5.0%, the result will be unreliable and absolute inhibition occurs at 20.0% blood contamination.^18^ However, studies found that patients who are MTB positive in a blood sample have a higher risk of death.^36^ Therefore, we are missing the most important sputum quality. The current finding is different from a study in Uganda, where SNPT was more in salivary sputum and lowest in blood-stained sputum.^20^ This might be as a result of the fact that the majority of the Ugandan study participants were HIV positive and most of them had a very low CD4 count (≤ 200 cells/µL). The second highest SNPT positivity, 7.7%, was diagnosed from purulent sputum. Although the difference is not statistically significant, blood-stained sputum showed greater positivity than purulent sputum, whereas purulent sputum is considered the best sputum for MTB detection. The difference in macroscopic sputum appearance significantly varied for MTB positivity by Xpert^®^ MTB/RIF test, implying a need for proper sputum collection, similar to other reports.^20,33,34^

In Ethiopia, the sputum sample collection strategy for MTB diagnosis was changed from spot-morning-spot to spot-spot (same-day diagnosis) in 2017.^26^ Same-day diagnosis stops multiple visits to the health facilities by the patient to submit sputum and receive a result. However, it is 2.8% less sensitive with a lower dropout rate than the conventional -spot-morning-spot strategy.^21^ In the spot-morning-spot strategy, three smear slides are made. The first slide made from the first spot sputum, the second slide made from the morning sputum and the third slide from the second spot sputum.^22^ This change increases the possibilities of being smear-negative. In the spot-spot approach, in the current study, Xpert^®^ MTB/RIF assay detected an extra 24 (5.7%) SNPT and three RR strains in comparison with smear microscopy. The ability of the Xpert^®^ MTB/RIF test to detect SNPT and RR is high in this study which might be because the missed morning sputum increased the SNPT cases. The Xpert^®^ MTB/RIF testing was performed on the direct sputum before culture to evaluate the performance of the Xpert^®^ MTB/RIF in the peripheral health facilities where direct sputum only is used for Xpert^®^ MTB/RIF testing. Sputum processing, including decontamination, neutralisation, and pellet concentration before culture, is only possible in the tuberculosis culture reference laboratories.

The diagnostic sensitivity of the Xpert^®^ MTB/RIF test in this study was 92.3%, while specificity was 99.2% reference to tuberculosis culture. The present study revealed higher sensitivity and specificity than the Uganda report^20^ because most of Uganda’s study participants were HIV-positive and used a spot-morning-spot diagnostic approach. Another justification might be tuberculosis prevalence is higher in Ethiopia. Similarly, the present study revealed a very high sensitivity compared to a study in Jigjiga, Ethiopia: 48.5% for smear-negative PTB.^37^ Likewise, the current study showed higher sensitivity and specificity relative to a review conducted in Liverpool, United Kingdom, in 2013, which was 67.0% – 74.0% pooled sensitivity and 99.0% pooled specificity.^38^ However, the sensitivity of the Xpert^®^ to diagnose SNPT showed considerable variability in different studies.^39,40,41^ The possible reasons for the variabilities in sensitivity and specificity might be differences in study design, study population, sample collection strategy, study period, study area or location, tuberculosis prevalence, comorbidity and laboratory performance. The three (11.54%) RR strains detected by the Xpert^®^ MTB/RIF test in this study concords 100.0% with Genotype MTBDR plus version 2 and phenotypic BACTEC™ MGIT™ 960 DST results. In addition, studies reported high Xpert^®^ MTB/RIF detection performance: 95.0% – 97.0% sensitivity and 98.0% – 99.0% specificity, for culture positives.^38^

The findings in this study are commendable since they facilitate early tuberculosis diagnosis and universal access to DST,^42^ which are pivotal to the endTB strategy. Furthermore, the World Health Organization approved that tuberculosis diagnostic technologies such as Xpert^®^ MTB/RIF need to be scaled up to become the first line of diagnosis. With the scale-up, patients will benefit from early diagnosis and initiation of treatment.

More than half of the positive Xpert^®^ MTB/RIF results (14; 51.9%) had a low or very low bacillary load, implying that SNPT patients have a paucibacillary load. Twenty (4.8%) Xpert^®^ MTB/RIF results were unsuccessful (the sum of errors, invalids and no results), which is lower than a report from Nigeria.^43^ The Nigeria study did not include only presumptive smear-negative SNPT cases. Most of our unsuccessful results were due to error results (15; 3.6%), but in the Nigerian study, these were considered invalid results.^43^ The error rate in the present study was higher than the GLI recommendation (< 3.0%),^44^ but lower than a report from Addis Ababa, which reported 8.9%.^45^ The discrepancy in the study reports might result from the difference in the testers’ expertise and experience, study population, sample type, and study period. The error code 5007 was the most common (12; 2.9%), often caused by viscous sputum or wrong sample volume, improper filling of cartridge reaction tube, bubbles, or probe integrity issues. Errors are a loss of valuable time and money for the patients and the laboratory. Thus, patient training on quality sputum collection and laboratory staff refresher training may minimise these errors. Of the three RR cases detected by the Xpert^®^ MTB/RIF test, two were due to probe E (codons 529–533) missing, which is the most frequent type of mutation in the rpoB region of the mycobacteria.^46,47^ The other was probe B missing (codons 511–518; mutation).

### Limitations

The current study did not explain the performance of the Xpert^®^ MTB/RIF test in the same-day diagnosis to diagnose smear-negative RR as it did not include enough RR cases, which is a limitation of the study.

### Conclusion

The performance of the Xpert^®^ MTB/RIF test to detect SNPT in spot-spot samples and rapid detection of RR were high. However, the diagnostic performance of the test significantly varied across different sputum qualities. Thus, good patient instruction or training and close follow-up on sputum sample collection are essential for getting quality sputum for better Xpert^®^ MTB/RIF yield. We recommend testing sputum samples by Xpert^®^ MTB/RIF irrespective of sputum quality.
